# Anti-arrhythmic properties of non-antiarrhythmic medications

**DOI:** 10.1016/j.phrs.2020.104762

**Published:** 2020-06

**Authors:** Emmanuel Ato Williams, Vincenzo Russo, Sergio Ceraso, Dhiraj Gupta, Richard Barrett-Jolley

**Affiliations:** a Department of Cardiology, Liverpool Heart and Chest Hospital, Thomas Drive, Liverpool, L14 3PE, United Kingdom; b Chair Neuropharmacology, Institute of Aging and Chronic Disease, University of Liverpool, United Kingdom; c Chair of Cardiology, Department of Medical Translational Sciences, University of Campania "Luigi Vanvitelli", Monaldi Hospital, Naples, Italy; d Specialization Fellow in Cardiology, Department of Medical Translational Sciences, University of Campania "Luigi Vanvitelli" - Monaldi Hospital, Naples, Italy; e Institute of Aging and Chronic Disease, University of Liverpool, United Kingdom

**Keywords:** Antiarrhythmic, Arrhythmia, Nti-arrhythmic, Statins, PUFA, ACE

## Abstract

Traditional anti-arrhythmic drugs are classified by the Vaughan-Williams classification scheme based on their mechanisms of action, which includes effects on receptors and/or ion channels. Some known anti-arrhythmic drugs do not perfectly fit into this classification scheme. Other medications/molecules with established non-anti-arrhythmic indications have shown anti-arrhythmic properties worth exploring.

In this narrative review, we discuss the molecular mechanisms and evidence base for the anti-arrhythmic properties of traditional non-antiarrhythmic drugs such as inhibitors of the renin angiotensin system (RAS), statins and polyunsaturated fatty acids (PUFAs).

In summary, RAS antagonists, statins and PUFAs are ‘upstream target modulators’ that appear to have anti-arrhythmic roles. RAS blockers prevent the downstream arrhythmogenic effects of angiotensin II – the main effector peptide of RAS – and the angiotensin type 1 receptor. Statins have pleiotropic effects including anti-inflammatory, immunomodulatory, modulation of autonomic nervous system, anti-proliferative and anti-oxidant actions which appear to underlie their anti-arrhythmic properties. PUFAs have the ability to alter ion channel function and prevent excessive accumulation of calcium ions in cardiac myocytes, which might explain their benefits in certain arrhythmic conditions.

Clearly, whilst a number of anti-arrhythmic drugs exist, there is still a need for randomised trials to establish whether additional agents, including those already in clinical use, have significant anti-arrhythmic effects.

## Graphical abstract

**Figure:**
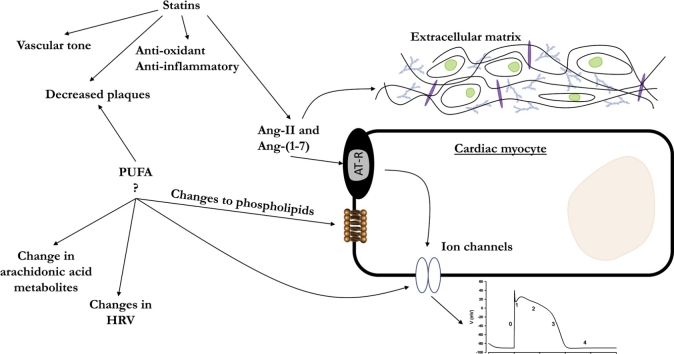

## 1 Introduction

Anti-arrhythmic drugs (AADs) modulate the activity of ion channels and receptors in the heart. The original Vaughan-Williams classification does not include drugs such as those belonging to the recently proposed class 0 (e.g. ivabradine) [which reduces the sino-atrial node (SAN) automaticity by blocking the pacemaker channel underlying the “funny current” (*I*_f,_ largely HCN4) [1]. Modification of the original classification scheme has become necessary as newer AADs (such as ivabradine, vernakalant, etc) and anti-arrhythmic properties of non-antiarrhythmic medications increasingly become apparent. In this review, we examine the evidence for *anti-arrhythmic* properties of drugs not traditionally thought of as antiarrhythmic.

### 1.1 Normal cardiac electrical activity

Cardiac activity relies on its action potential and excitation-contraction coupling. A normal cardiac impulse is generated by the pacemaker cells in the SAN, conducted through the atrioventricular node (AVN), and the His-Purkinje network (HPN) to facilitate activation of the ventricles. Cardiac arrhythmias occur from any abnormalities affecting electrical impulse generation, conduction or both. The SAN, AVN, HPN, coronary sinus (CS) and pulmonary veins (PVs) are capable of spontaneous electrical impulse generation (automaticity) underpinned by spontaneous diastolic depolarisations during phase 4 of the cardiac action potential; although the SAN sets the pace under normal physiological conditions with its pacemaker potential [1].

**Fig. 1:**
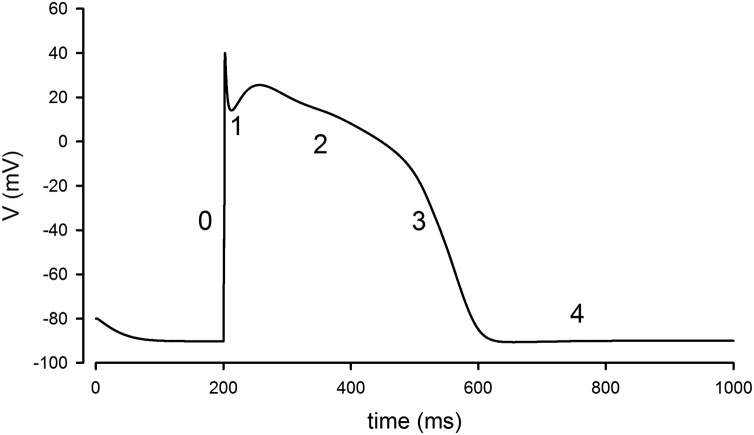
**The ventricular action potential**. Ventricular action potential simulated in python NEURON [150] using an adaptation of the DiFrancesco and Noble model [151] and stimulating with a 2 nA current injection at time 0.2 s. The four phases of the action potential are illustrated on the waveform. Phase 0 is the upstroke of the action potential resulting from the large rapid sodium (Na^+^) current, activated once the activation threshold is exceeded. Phase 1 occurs from the inactivation of the Na^+^ current while there is activation of a transient outward potassium (K^+^) current. Phase 2 is the plateau largely resulting from a balanced inward calcium (Ca^2+^) and outward delayed rectifier (K^+^) current. Phase 3, the downward stroke, occurs as the Ca^2+^ inactivates whilst the delayed rectifier current persists. In a ventricular myocyte, by phase 4 the cell has returned to the resting membrane potential and the voltage-gated currents will “reset” (recover from inactivation), ready for the next action potential. A key difference in nodal tissues (e.g. sinoatrial node) is that phase 4 of the nodal action potential (not shown) is a period of spontaneous depolarisation. Some established anti-arrhythmic drugs modulate specific phases of the action potential by their effects on specific ion currents e.g. Na^+^ (quinidine, lidocaine, mexiletine, flecainide) and K^+^ (amiodarone, sotalol, dofetilide). For instance, amiodarone modulates the hERG (human Ether-à-go-go-Related Gene) K^+^ channel that controls action potential duration [152].

Ion fluxes at different phases of the cardiac potential determine whether there is resulting depolarisation or repolarisation (Fig. 1).

**Fig. 2:**
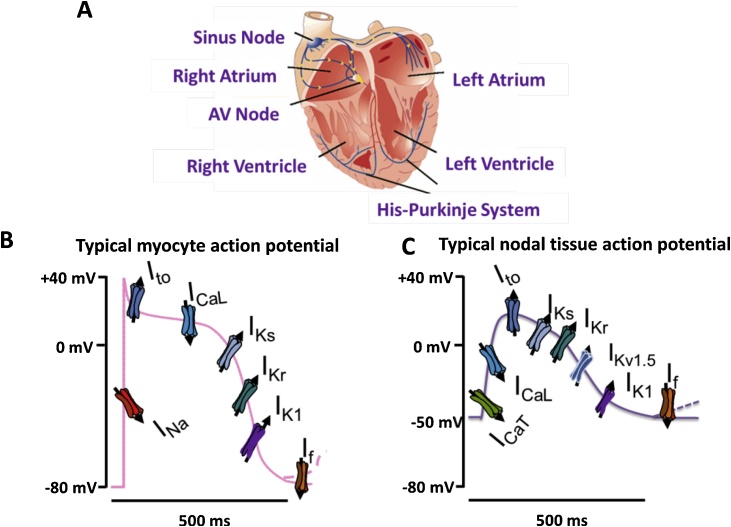
**Illustration of cardiac action ion channel involvement in cardiac action potentials. (A)** The basic cardiac structure with annotated conduction pathway from SAN to AVN via the His-Purkinje pathway. **(B)** The main ion channels involved with cardiac muscle action potentials, ventricular and atrial myocyte action potentials are similar; although typically the ventricular myocyte will have a broader plateau. **(C)** The nodal tissue, i.e., sinoatrial (SAN) and atrioventricular (AVN), are also similar with less pronounced spike and less negative resting membrane potential. Current (other name): Gene, Protein [additional genes/proteins]. I_Na_: *SCN5A*, Nav1.5. I_to_ (fast): *KCND2*, Kv4.2 [*KCND3*, Kv4.3, *KCNIP2*, KChIP2]. I_to_ (slow): *KCNA4*, Kv1.4, I_Ca-L_: *CACNA1C*, Cav1.2: I_Ks_, *KCNQ1*, Kv7.1 [*KCNE1*, mink]. I_Kr_: *KCNH2*, Kv11.1 [*KCNE2*, MiRP-1]. I_K1_: *KCNJ2*, Kir2.1 [*KCNJ12*, Kir2.2, *KCNJ4*, Kir2.3]. I_f_ : *HCN4*, HCN. ICaT: *CACNA1G* Cav3.1 [*CACNA1H*, Cav3.2]. Kv1.5: *KCNA5*, Kv1.5. Figure from [153] With permission of Elsevier.

There has been significant progress made in delineating the ion fluxes underlying the different phases of the human cardiac action potential since early attempts by electrophysiologists in the 1900′s using frog, sheep, calf and turtle myocardial models [2]. An initial depolarisation (*phase 0*) results from a large rapid flux of sodium current (*I*_Na_) carried by the cell membrane’s fast activating sodium channels when the depolarisation threshold is exceeded. *Phase 1*, which follows, consists of minor repolarisation resulting from non-conducting inactivated sodium channels and the transient outward potassium current (*I*_to_). The plateau (*phase 2*) results from a depolarising inward calcium flux carried by the L-type calcium (Ca) channels (which is induced by the initial inward sodium current) and a repolarising outward potassium current. Depolarisation of atrial myocytes activates a transient outward potassium current which rapidly inactivates, leading to a sustained outward current. The sustained current in atrial myocytes is due to activation of an ultra-rapid delayed rectifier potassium current (*I*_Kur_) [3], [4]. The outward potassium conductance that persists throughout the plateau phase to effect repolarisation is referred to as the ‘*delayed rectifier*’ current. *Phase 3* repolarisation is due to inactivation of the calcium current with persistence of the *rapid* and *slow* components of the delayed rectifier potassium current (*I*_Kr_ and *I*_Ks_). *Phase 4* is mediated by multiple potassium channels which carry the repolarising potassium current. These include the *transient outward* potassium current (*I*_to_), and the delayed rectifier potassium current (*I*_Kr_ and *I*_Ks_) [4]. The potassium current (I_K1_) establishes the resting membrane potential of the myocyte during this phase. *Spontaneous diastolic* (*phase 4*) *depolarisation* in cells capable of automaticity (such as nodal cells) is believed to be generated by activation of the inward *I*_f_ current during diastole [5]. Other currents including the potassium and calcium currents, potassium ATPase (*I*_KATP_) and the sodium-calcium exchanger (*I*_NCX_), etc. may play roles in spontaneous diastolic depolarisation, but the extent of such roles need further clarification. More recent molecular characterisation has now largely, but not entirely led to identification of a number of ion channels important for each phase of the action potential (Fig. 2).

## 2 Angiotensin-Converting enzyme inhibitors/ angiotensin receptor blockers

The Renin-Angiotensin System (RAS) is an established key player in the regulation of fluid and electrolyte balance and blood pressure. Angiotensin II (Ang II) is the main effector peptide of RAS, acting primarily via angiotensin II type 1 (AT1R) and 2 receptors (AT2R) [6]. Angiotensin Converting Enzyme (ACE) is a central component of RAS (given its role in the generation of Ang II), and is a principal target in the management of cardiovascular (hypertension, heart failure, post myocardial infarction) and renal diseases. Ang II, acting on AT1R, ultimately leads to effects such as vasoconstriction, aldosterone and antidiuretic hormone (ADH) release, oxidative stress, hypertrophy, proliferation, and fibrosis [7]. In addition to the established effects, activation of RAS has been suggested to play a role in the generation and maintenance of arrhythmias such as atrial fibrillation (AF), as it modulates certain ion currents thought to be involved in the pathogenesis of the arrhythmia [8]. Blockade of RAS with Angiotensin Converting Enzyme Inhibitors (ACE-Is) and/or Angiotensin Receptor Blockers (ARBs), therefore, appears promising as a therapeutic target in the management of certain arrhythmic conditions.

### 2.1 Molecular mechanisms of anti-arrhythmogenicity

Anti-arrhythmic roles for ACE-Is and AT1R blockers appear plausible on consideration of the molecular effects of some components of RAS. Chronic AF is associated with the down-regulation and up-regulation of AT1 and AT2 in human atrial tissue, respectively [9]. Similarly, interstitial fibrosis increases the levels of the AT2 receptor - mainly expressed on cardiac fibroblasts - where it opposes Ang II-induced hypertrophic signals [10]. Ang II and Ang-(1−7) themselves have a number of effects on cardiac ion channels critical to maintenance of cardiac rhythm (Table 1) together with potential indirect effects via modulation of the extracellular matrix [11].

**Table 1:** Ang-II and Ang-(1-7) action actions on ion channels.

Target	**Action**	**References**
***I*_Ks_**	Chronic *in vivo* Ang II exposure increases ***I*_Ks_** in atrial myocytes, while decreasing them in ventricular myocytes.	[12]
***Kv4.3 / I*_to_**	Ang II can alter the current density of *I*_to_ in myocyte membranes. (1) Downregulation by internalisation, where angiotensin II receptor type 1 (AT1R) colocalises with Kv4.3, to form a molecular complex that is internalised via the well-established phenomenon of AT1 endocytosis. (2) Modulation of gating properties of Kv4.3; such that the Kv4.3 activation voltage threshold is increased/decreased.	[13,14,15]
***I*_CaL_**	The L-type Ca channel current (***I*_CaL_**) is increased in atrial myocytes after chronic exposure to Ang II, which contributes to plateau elevation of the action potential and prolongation of the APD.	[12]
***I*_ti_*, I*_K_**	Ang II also increases the delayed rectifier potassium (*I*_K_), transient inward (*I*_ti_), pacemaker, and sodium-calcium exchanger (*I*_NCX_) currents in pulmonary vein cardiomyocytes, whilst AT1 antagonists, such as losartan, decrease the *I*_to_, *I*_k_, *I*_ti_, and *I*_NCX_ currents [8].	
***I*_Na_**	Ang-(1−7) significantly increases the cardiac sodium current (*I*_Na_) densities, contributing to improved intra-atrial conduction, which reduces the likelihood of re-entry (and therefore decreases likelihood of arrhythmia induction and maintenance).	[16,17]

RAS could also influence arrhythmogenicity via modulation of extracellular matrix protein expression and cardiac remodelling. Ang II leads to proliferation, while Ang-(1−7) leads to anti-proliferation. Progressive accumulation of fibrotic tissue in the myocardium is a major contributor to structural cardiac remodelling, along with dilatation and myocardial hypertrophy. Structural remodelling includes changes in both the cellular components (myofibroblasts, fibroblasts) and the extracellular matrix. Ang II has direct proliferative effects on atrial and ventricular fibroblasts and smooth muscle cells [11]. Ang II is also a potent stimulator of collagen synthesis by cardiac fibroblasts [18]. It promotes cellular growth and hypertrophy through the activation of mitogen-activated protein kinases (MAPKs). Ang II also promotes the expression of other profibrotic factors such as endothelin 1, ET-1 [18], while the downstream generation of aldosterone is also pro-fibrotic by direct or indirect stimulatory effects on fibroblasts or macrophages, respectively [19]. Chronic persistent and paroxysmal atrial fibrillation are associated with increased ACE activity, along with increased activated ERK-1/ERK-2 and the ERK activating kinases (MEK 1/MEK2) in the interstitial cells associated with marked atrial fibrosis.

### 2.2 Evidence from pre-clinical studies

AF is the commonest type of arrhythmia; and it is associated with remodelling (electrical and structural), which facilitate the occurrence of the arrhythmia. In many cases, the electrical remodelling is thought to be mediated by rate-induced intracellular calcium overload in the short term [20,21], and include reductions in: 1) the APD [22], 2) the atrial effective refractory period (ERP) [20,[22], [23], [24], [25]], 3) the ERP accommodation to heart rate [17,22], and 4) atrial conduction velocity (CV) [[22], [23], [24], [25]]; and prolonged 5) inter-atrial conduction time and 6) AF duration [26]. Reduced APD and ERP are generally regarded as important factors in re-entry-based AF [27]. Ang II/AT1 may play important roles in electrical remodelling, and their inhibition by ACE-Is/AT1R blockers may be preventive of such remodelling [25,28], as shown with candesartan [28], captopril [28], and irbesartan [27], [29,30]. Structural remodelling includes tissue fibroses [26], dilatation and hypertrophy. Mechanical stretch in response to haemodynamic load causes the release of Ang II which essentially feeds-forward to produce a hypertrophic response [31]. Ang II enhances synthesis of extracellular matrix collagen via both AT1R and AT2R [32]. Electrical heterogeneity from tissue fibrosis is thought to be a critical factor leading to the induction and promotion of arrhythmia [21], [33].

Chronic rapid atrial activation (by pacing) promotes the induction of sustained AF. In a canine model, this was shown to occur from a reduction in both the sodium current (*I*_Na_) density and the atrial conduction velocity [17]. Similarly, Ang II leads to a reduction in transcription of the sodium channel (SCN5A) and the *I*_Na_ by promoting the formation of hydrogen peroxide (H_2_O_2_), which instigates the binding of nuclear factor kappa B (NF-κB) to the SCN5A promoter [34]. RAS inhibition with ACE-I or AT1R blocker prevents the down-regulation of the SCN5A and the *I*_Na_ [17], which can reduce the pro-arrhythmic risk in conditions with increased Ang II activity such as AF and heart failure (HF) [25].

Ang II plays a critical role in the induction of ventricular arrhythmias during ischaemia-reperfusion injury. This was demonstrated with the observation that the number of premature ventricular complexes (PVCs) in AT1R knock-out mice was much less compared to the number in wild-type controls in ischaemia-reperfusion injury; and that selective treatment with an AT1R blocker in wild-type mice prior to ischaemia ameliorated the occurrence of the PVCs [35].

Reduction in atrial fibrosis may also contribute to reduction in structural remodelling, and hence modulation of the pro-arrhythmic substrate. By virtue of their antifibrotic properties, RAS inhibitors are believed to play a role here by reducing the synthesis of collagen I molecules as well as promoting the degradation of collagen I fibres already formed [30], and reducing the expression of TGF-β [26].

### 2.3 Evidence from randomised trials

Potential beneficial effect of RAS inhibition on arrhythmias (e.g. AF) could be direct or secondary to neurohumoral modulation and antihypertensive effects. RAS inhibition has also been associated with indirect anti-arrhythmic effects – as identified for some clinical trials set-up for non-arrhythmic primary outcomes (i.e. observational effects or secondary outcome measures). Randomised controlled trials are weightier measures on the evidence scale; hence, a few trials have sought to prove a direct anti-arrhythmic effect for RAS inhibitors with rather mixed results (Table 2).

**Table 2:** Randomised Trial data for RAS inhibition on arrhythmias.

**Study**	**Cohort size**	**Headline**	**Refs**
TRAndolapril Cardiac Evaluation (**TRACE**)	1577	The study showed a significant difference in the development of AF in favour of the ACE-I.	[36]
In-hospital AF or flutter in the **GISSI-3** trial	17944	Reduction in AF post-AMI.	[37]
Congestive Heart Failure (CHF) arrhythmias	374	Significant reduction in the frequency of ventricular arrhythmias such as PVCs, ventricular couplets and VT.	[38]
Studies Of Left Ventricular Dysfunction (**SOLVD**)	55	Significantly fewer patients with AF in enalapril group.	[39]
Valsartan Heart Failure Trial (**Val-HEFT**)	4395	Significantly lower AF incidence in patients with HF randomised to either valsartan or placebo on top of HF treatment.	[40]
Candesartan in Heart failure: Assessment of Reduction in Mortality and morbidity **(CHARM)**	392	Candesartan reduced incidence of AF in patients with symptomatic HF.	[41]
Heart Outcomes Prevention Evaluation **(HOPE)**	8335	Over 4.5 years of follow-up ramipril (compared to placebo) did not significantly reduce the incidence of AF in patients without known HF or left ventricular systolic dysfunction.	[42]
Valsartan Antihypertensive Long-term Use Evaluation **(VALUE)**	15245	Valsartan-based treatment of hypertension reduced the incidence of new-onset/sustained AF compared with an amlodipine-based treatment in hypertensive patients.	[43]
Gruppo Italiano per lo Studio della Sopravvivenza nell'Infarto Miocardico–Atrial Fibrillation **GISSI-AF** trial	1442	Valsartan had no effect on the recurrence rate of AF in patients with a history of AF (including post successful cardioversion).	[44]
Ongoing Telmisartan Alone and in Combination with Ramipril Global Endpoint Trial **(ONTARGET)**	25577	No changes in new onset AF in combining telmisartan and ramipril, but an increased risk of adverse effects.	[45]
Atrial Fibrillation Clopidogrel Trial with Irbesartan for Prevention of Vascular Events **(ACTIVE 1)**	9016	Irbesartan did not significantly reduce the risk of hospitalisation of patients AF.	[46]
Japanese Rhythm Management Trial II for Atrial Fibrillation **(J-RHYTHM II)**	326	Candesartan, combined with amplodipine, gave no advantage of amplodipine alone in terms of paroxysmal AF frequency.	[47]

The TRAndolapril Cardiac Evaluation (**TRACE**) study generated very promising results. Patients with reduced left ventricular function (LVF) secondary to acute myocardial infarction (AMI) in sinus rhythm (SR) received either trandolapril or placebo and were followed up for 2–4 years for the primary outcome measure of the development and time to occurrence of AF in one 12-lead ECG recorded at an outpatient visit. The study showed a significant difference in the development of AF in favour of the ACE-I with a relative risk of approximately 50 % albeit with a broad confidence interval) in patients with left ventricular dysfunction (LVD) secondary to AMI (95 % CI: 0.26−0.76 [36]. In larger trial (**GISSI-3)** there was a 24 % reduction in AF incidence (OR 0.76; 95 % CI: 0.65−0.89) in patients with both an ACE-I (lisinopril) and nitrates compared to controls [37]. Further positive data showed that enalapril, given alongside maintenance therapy with digoxin and furosemide in Congestive Heart Failure (CHF) (New York Heart Association functional class II-III) significantly reduced the frequency of ventricular arrhythmias such as PVCs, ventricular couplets and VT [38]. A further retrospective study (**SOLVD**) involving patients with LVD in SR revealed a significant difference in the incidence of AF in patients who had received enalapril compared to those who received placebo after mean follow up of nearly 3 years. On multivariate analysis, enalapril was the most powerful predictor for risk reduction of AF (hazard ratio, HR, 0.22; 95 % CI: 0.11−0.44) [39]. Similarly, results from sub-analyses of the Valsartan Heart Failure Trial (**Val-HEFT**) showed a significant difference in AF incidence in patients with HF randomised to either valsartan or placebo on top of HF treatment. [40]. A meta-analysis of three ARB studies found a significant beneficial effect of RAS inhibition on the occurrence of AF in the setting of HF (OR 0.52; 95 % CI: 0.31−0.87) [48]. In contrast the **HOPE** trial, however, found that over 4.5 years of follow-up ramipril (compared to placebo) did not significantly reduce the incidence of AF in patients without known HF or left ventricular systolic dysfunction (LVSD) [42]. Studies suggests the beneficial effects of RAS inhibitors, such that they are, in preventing AF are not just secondary benefits from improved haemodynamics such as controlled BP [49,43].

However, a number of studies are less promising; in the **GISSI-AF** trial, valsartan was not associated with a reduction in the incidence of recurrent AF [44] and treatment with candesartan before and after electrical cardioversion also had no effect on the recurrence rate of AF [50]. Furthermore, other trials including **ONTARGET**, **ACTIVE I**, and **J-RHYTHM II** have found little additional beneficial effect against AF recurrence using RAS blockade along with an AAD (e.g. amiodarone, sotalol, propafenone) [51,[45], [46], [47]].

## 3 Statins

Statins are important medications with established indications in primary and secondary prevention of coronary heart disease. They decrease the cellular cholesterol content by selectively inhibiting the rate-limiting enzyme of cholesterol synthesis pathway (also known as ‘mevalonate pathway’): 3-hydroxy-3-methylglutaryl-coenzyme A (HMG-CoA) reductase. As well as limiting cholesterol biosynthesis, they lower serum and hepatic cholesterol and serum triglyceride concentrations.

### 3.1 Molecular mechanisms

A counter-regulatory mechanism of reduced cholesterol biosynthesis is upregulation of low-density lipoprotein (LDL) receptor expression in the hepatocyte membrane, with accelerated hepatic clearance of circulating LDL cholesterol [52]. Furthermore, statins show some additional pharmacodynamic (‘pleiotropic’) effects that are inexplicable with the lipid-lowering mechanism alone [53]. A number of these pleiotropic effects are ultimately cardioprotective.

By reducing the production of mevalonate (a key intermediate compound during cholesterol biosynthesis) through the inhibition of HMG-CoA reductase, statins impair the downstream production of isoprenoid intermediates (e.g. farnesylpyrophosphate and gerany-geranylpyrophosphate). This implies inhibition of post-translational modifications (e.g. transferase-catalysed addition of farnesyl and geranylgeranyl isoprenoids to small hydrolase enzymes, such as Ras and Ras-like proteins (e.g. Rho and Rac), that hydrolyse guanosine triphosphate, i.e. GTPases [54]). Rho and its downstream target, Rho-associated coiled-coil-containing kinase (ROCK), are mediators of endothelial dysfunction [55], hence their inhibition can improve endothelial function, and decrease vascular inflammation and atherosclerosis [56]. Endothelial Peroxisome Proliferator-Activated Receptor-gamma (PPAR-γ) protects against oxidative stress and endothelial dysfunction by suppressing the activity of Rho and ROCK [57]. Statin inhibition of Rho and ROCK is a key underlying mechanism mediating some of the pleiotropic effects [56,58],^59^]. Furthermore, the Rho/ROCK pathway contributes to myocyte apoptosis and hypertrophy and interstitial fibrosis occurring as part of pathological cardiac hypertrophy secondary to Ang II [55,56]. The mechanisms underlying some pleiotropic effects and potential anti-arrhythmic effects of statins are listed in Table 3.

**Table 3:** Mechanisms underlying pleiotropic and potential anti-arrhythmic effects of statins ROCK: Rho-associated coiled-coil-containing kinase; eNOS: endothelial Nitric Oxide Synthase; PI(3)K: Phosphatidylinositol-3-OH kinase; Akt: protein kinase B; mRNA: messenger ribonucleic acid; MMP: Matrix Metalloproteinase; GTPase: Guanosine Triphosphate Phosphohydrolase; NADPH: Nicotinamide Adenine Dinucleotide Phosphate Hydrogen; SMC: Smooth Muscle Cell; IL- Interleukin; TNF-α: Tumour Necrosis Factor-alpha; CRP: C-reactive protein; TGF-β: Transforming Growth Factor – beta; NF-κB: Nuclear Factor-kappa B; MAPK: Mitogen-Activated-Protein Kinase; HCM: Hypertrophic Cardiomyopathy.

Effect	Mechanism	References
**Improved vascular tone:**		
Up-regulation of endothelial nitric oxide synthase (eNOS)	Inhibition of ROCK (which downregulates eNOS)	[59,81]
	Activation on PI(3)K/Akt pathway (which increases eNOS activity)	[68,82,83]
	Post-transcriptional eNOS mRNA stabilization	[67]
Increase in endothelial progenitor cells	Activation on PI(3)K/Akt pathway	[84,85]
	Inhibition of endothelin 1	[86]
Improved endothelial function	Inhibition of superoxide formation	[64]
**Reduced plaque vulnerability**	Reduction of arterial wall myocyte migration and proliferation	[87]
	Inhibition of macrophage cholesterol esterification	[88]
	Inhibition of matrix metalloproteinase (e.g. MMP-2, MMP-9, MMP-12) secretion	[89,90]
**Anti-oxidant effects:**		
Inhibition of Ang II-induced superoxide formation in myocytes and vascular SMC	Inhibition of GTPase Rac1 required for NAD(P)H oxidase activity	[91]
	Reduced mRNA expression of NADPH oxidase subunits (Nox1, p22phox)	[92]
**Anti-inflammatory effects**	Reduced stimulation of pro-inflammatory cytokines (e.g. TNF-α, IL-1β, IL-6, IL-8, etc.) and CRP	[93]
	Inhibition of RhoA-mediated TNF-α-induced NF-κB activation	[94]
**Attenuation of Ang II-mediated cardiovascular remodelling**	Inhibition of Rac1-mediated NADPH oxidase activity in vascular SMCs and heart	[91]
	Reduction of activated Ras and MAPK in a transgenic model of human HCM	[95]
	Inhibition of TGF-1β-Smad 2/3 signalling pathway	[96]

Briefly, the possible underlying mechanisms of anti-arrhythmic effects of statins include:

- atherosclerotic plaque stabilisation and regression [60,61], (i.e. improved myocardial perfusion and less risk of ischaemia-reperfusion or scar-related arrhythmia, etc.),
- inhibition of oxidised LDL (oxidised LDL upregulates AT1R possibly via a nuclear factor kappa B- NFκB-dependent pathway) [62,63],
- anti-oxidant, anti-inflammatory and anti-proliferative effects [[64], [65], [66]],
- regulation of nitric-oxide-dependent coronary arterial tone and endothelial function [67,68],
- attenuation of arrhythmogenic structural remodelling via mechanisms including upregulation of transcription factor GATA-6 expression and inhibition of the Rho pathway [[69], [70], [71], [72]],
- improvement in the heterogeneity of cardiac repolarisation [73] (i.e. QT dispersion, QTd – increased QTd is associated with the incidence of life-threatening ventricular arrhythmias [74,75]);
- increase in heart rate variability, HRV (decreased HRV is believed to be associated with reduced parasympathetic tone and predicts arrhythmic events [76,77]) and
- changes in transmembrane ion channel conductance [[78], [79], [80]].

### 3.2 Statins and ventricular arrhythmias

**MADIT-CRT** (Multicentre Automatic Defibrillator Implantation Trial with Cardiac Resynchronization Therapy) was a randomised trial that examined the potential anti-arrhythmic benefit of statin use in non-ischaemic cardiomyopathy (NICM). Statin use was associated with a significant (77 %) decrease in the risk of cardiac death or life-threatening ventricular arrhythmias (VT/VF) [97]. Similarly, **MADIT-II** trial also showed beneficial anti-arrhythmic effect of statin use in implantable cardioverter-defibrillator (ICD) patients by lowering the risk of VT/VF and cardiac death [98]. Statin use in patients with atherosclerotic heart disease (ASHD) treated with ICD, led to decreased probability (40 % decrease in relative hazard) in the recurrence of VT/VF in the Antiarrhythmics Versus Implantable Defibrillators (**AVID**) trial [99]; while in the Global Registry of Acute Coronary Events (**GRACE**), there was association with reduced VT, VF or cardiac arrest (as well as AF) [100]. Furthermore, the Thai Registry of Acute Coronary Syndrome (**TRACS**) – associated statins with decreased incidence of ventricular arrhythmias (VA) [101].

In summary, statins appear to show beneficial protective effect against life-threatening ventricular arrhythmias. The possible mechanisms underlying this protective effect may be related to effects including improved coronary artery tone, atherosclerotic plaque stabilisation, anti-inflammatory, anti-oxidative, and improved cardiac repolarisation heterogeneity [79].

#### 3.2.1 Statins and atrial fibrillation

Inflammation and oxidative stress have been of key interest among the possible mechanisms that underlie AF pathogenesis. In acute inflammatory states, such as cardiac or non-cardiac surgery and myo-pericarditis, new-onset AF coincides with peak levels of inflammatory biomarkers [102]. Theoretically, statins can protect against AF by reducing the burden of vascular disease and by mitigating atrial remodelling via pleiotropic anti-inflammatory, anti-oxidative, anti-proliferative and antithrombotic effects; as well as by improvement of endothelial function and neurohormonal regulation. Statins may act through these pleiotropic effects in reducing AF morbidity and mortality [79].

Statins decreased the risk of new-onset AF by 19 % - more so with CHADS_2_ score ≥ 2 than CHADS_2_ = 1 (Taiwan’s National Health Insurance research database) [103]. On the contrary, Cabratosa-Alves et al., reported only minimal protective effect of statins against new-onset AF in lone hypertension in a Spanish registry [104].

A meta-analysis by Fang et al., showed an overall significant reduced risk of AF incidence/recurrence [105].

Inflammation and abnormal oxidative stress may be the main pathophysiological features related to atrial remodelling and enhanced myocardial tissue inflammation, resulting in AF onset, recurrence, and persistence [106]. Statins are well-known to reduce inflammation and oxidative stress - which could underlie their anti-arrhythmic effect [107].

## 4 Polyunsaturated fatty acids (PUFAs)

Polyunsaturated fatty acids (PUFAs) are best known as constituents of fish oils, but they are also found in soybean and certain rapeseed oils but not ordinary olive oil [108]. N-3 and n-6 PUFAs are involved in important cell homeostasis, contributing to plasma membrane structure, cell metabolism and response to oxidative stress and inflammation [109,110]. In an early study Kromhout et al. 1985 [111] reported that coronary heart disease of those eating substantial quantities of fish was half that of the control group. Since then, cardiovascular health benefits of n-3 PUFA have been widely reported in terms of coronary heart disease and sudden death in animal models of AF, but less so in terms of reduced human atrial fibrillation or ventricular arrythmia [112]. Broadly, there appears to be an inverse correlation between fish intake and incidence of AF [[113], [114], [115]], but a recent Cochrane systematic review of PUFA for prevention of cardiovascular disease reports that clinical evidence of beneficial effects of PUFA on atrial fibrillation is of very low quality [116].

### 4.1 Molecular mechanisms

Experimental studies show numerous potential molecular mechanisms by which PUFAs could potentially alter cardiac rhythmicity. At the level of the heart itself, PUFA exposure can alter myocardial membrane phospholipid composition and can, in turn, be anti-inflammatory or decrease potential Ca^2+^ overload [[117], [118], [119], [120], [121], [122]]. Either directly, or indirectly via such phospholipid changes, PUFA can decrease membrane excitability via alteration of ion channel activity (e.g., Na, K, L-type calcium) and currents (e.g., *I*_to_, *I*_k_, voltage-dependent sodium current) [[123], [124], [125], [126], [127]]. Ca2+ release through the ryanodine receptor is also decreased by PUFA [128] and there is an additional notable anti-VA potential of PUFA associated with connexin (cx43) mediate myocyte-myocyte coupling [128,129]. (Table 4).

**Table 4:** Possible mechanisms underlying anti-arrhythmic effect of polyunsaturated fatty acids *I*_to_: transient outward potassium current; *I*_k_: delayed rectifier potassium current; PUFA Polyunsaturated Fatty Acids; SR: Sarcoplasmic Reticulum; TXA_2_: Thromboxane A_2_; AA: Arachidonic acid; HRV: Heart Rate Variability; NEFA: Non-esterified Fatty Acids; cGMP: cyclic guanosine monophosphate; TGFβ1: Transforming Growth Factor β1; Akt: protein kinase B; EGF: Epidermal Growth Factor.

Effect	Mechanism	References
**Reduced substrate vulnerability**:		
Altered myocyte electrophysiology	Modulation of ion channel (e.g. Na, K, L-type calcium) conductivity and currents (e.g. *I*_to_, *I*_k_, voltage-dependent sodium current) leading to reduced myocyte excitability.	[123,124,125,126,127]
	Direct inhibition of SR calcium ion release channel/ryanodine receptor gating.	[128]
	Modulation of connexins.	[129,130]
Changes in myocardial membrane phospholipids	Insertion of n3-PUFA into cell membranes alters protein function and signalling: e.g. anti-inflammatory; anti-thrombosis – reduced platelet aggregation and adhesion via reduced production of TXA_2._ Altered cardiac myocyte membrane phospholipid composition: reducing n6-PUFA while increasing n3-PUFA is antiarrhythmic by reducing calcium ion availability Lowering of pro-arrhythmic membrane NEFA concentration, preventing intracellular calcium overload.	[117,118,119,120,121,122]
**Altered balance of AA metabolites**	Increased prostacyclin (anti-arrhythmic) to TXA_2_ (pro-arrhythmic) ratio.	[131,132]
**Improved HRV**	Calcium channel blocking effect on cardiac myocytes.	[133,134]
	Modulation of sympathetic nervous system.	[135]
**Reduction of trigger events and remodelling**		
Reduced atherosclerosis	Decreased plaque inflammation and increased plaque stability; reduced neovascularisation.	[136,137,138]
Reduced thrombosis	Reduced platelet aggregation.	[136]
Reduced cardiac fibrosis	Increase cGMP levels which inhibit TGFβ1-induced cardiac fibrosis by blocking phosphorylation and nuclear translocation of Smad2/3 as well as inhibitory effects on some structural remodelling signalling molecules (e.g. Akt, EGF).	[139,140],

### 4.2 PUFA and ventricular arrhythmias (VA)

Beginning in the 1980s, these fatty acids were documented to confer protection against arrhythmias in Wistar rats after coronary artery ligation-induced ischaemic injury [141]. Albert et al., also reported significant inverse correlation of n-3 PUFA levels with the risk of sudden death among men with no prior history of cardiovascular disease (i.e. high blood n3-PUFA associated with low risk of sudden death) [121]. The clinical data on potential benefit for VA are mixed, two trials in the same year (2005) reported apparently opposite results. Leaf et al., a marginally statistically significant reduction of life-threatening VA (relative risk reduction of 38 %) in an ICD population [142], but in similar study Raitt et al., found the opposite with increased risk of VT/VF in the n3-PUFA group [143]. This echoed the early work of Burr et al. 1989 challenging patients with increased consumption of fatty fish [144]. The differences in conclusions were striking since in both studies there were similar patient condition, cohort size, PUFA dose and change in the benchmark red blood cell membrane PUFA concentrations. The lack of anti-arrhythmic effects seen clinically in the latter study was very much supported by a more recent canine model where again n-3 PUFA were actually pro-arrhythmic under some conditions [145].

### 4.3 PUFA and atrial fibrillation (AF)

Despite the positive correlative data (above) linking reduced AF with high fish consumption, the clinical trials investigating whether n-3 PUFA protect against AF are again disappointing. In the large OPERA multicentre trial, fish oil failed to reduce post-operative AF [146] and a similar lack of efficacy of prescription n3-PUFA was reported for paroxysmal AF over a 6 month period [147]. AF is a common complication of coronary artery bypass graft surgery, but n3-PUFA supplementation also failed to reduce AF in this scenario too [148].

In summary there is still no consensus to what the anti-arrhythmic potential of PUFA really are, if any, despite approaching 40 years of investigation [149].

## 5 Future directions and conclusions

In summary, RAS blockers, statins and PUFAs appear to have beneficial roles in decreasing the incidence and recurrence of arrhythmias. The foregoing discussions illustrate that while some insight has been gained in the potential molecular pathways underlying their plausible anti-arrhythmic roles, further clarification of these mechanisms and additional direct evidence of antiarrhythmic effects through randomised controlled trials are necessary.

Only a few randomised trials to date have examined the potential anti-arrhythmic role of RAS blockers with no clear significant beneficial effects. However, there might still be some justification for future attempts aiming to identify direct anti-arrhythmic evidence through larger randomised controlled trials in patients with or without underlying cardiac disease. Future studies with statins could attempt to elucidate further the underlying molecular pathways explaining their pleiotropic effects. Furthermore, the identification of direct evidence for their anti-arrhythmic beneficial effects and the specific underlying pleiotropic effect at play are necessary. Further research into the differing effects of the major components of PUFAs (DHA and EPA), as well as clarification of the molecular mechanisms underlying their potential anti-arrhythmic roles are also required.

Based on the evidence examined above, it may not be possible to propose RAS blockers, statins or PUFA (EPA and DHA) are primary AADs. However, there appears to be strong molecular plausibility and evidence base supporting their use for obtaining adjunctive anti-arrhythmic benefits alone or in combination with already established AADs.

## Declaration of Competing Interest

None.
